# Supplementary material for: Transverse‐Electric Cherenkov Radiation for TeV‐Scale Particle Detection
Source: Adv Sci (Weinh). 2025 Oct 17;13(1):e13589. doi: 10.1002/advs.202513589 (PMC12767071; doi:10.1002/advs.202513589)
Supplement: Supplementary file 1 — Supporting Information [file ADVS-13-e13589-s001.pdf]

Supplementary Information for

“Transverse-electric Cherenkov Radiation for TeV-Scale Particle Detection”

Zhixiong Xie, Xiao Lin, Song Zhu, Chunyu Huang, Yu Luo, and Hao Hu

Supplementary Information Guide:

--Section S1. Dispersion relation of graphene plasmons in suspended graphene systems.

--Section S2. Calculation of the radiation pattern of Cherenkov radiation.

--Section S3. Calculation of the angular spectrum of Cherenkov radiation.

--Section S4. Influence of host materials on detectable momentum of Cherenkov detectors.

--Section S5. Radiation field pattern of TE graphene plasmon Cherenkov radiation.

--Section S6. More discussions on the influence of relaxation time on the radiation behaviors.

--Section S7. More discussions on the influence of chemical potential on the performance of particle detection.

--Section S8. More discussions on the influence of particle-graphene separation on the radiation intensity.

--Section S9. Detection scheme of TE graphene plasmon Cherenkov radiation.

--Section S10. More discussions on the low-index phonon polaritons operating in additional frequency band.

-- Supplementary References

Section S1. Dispersion relation of graphene plasmons in suspended graphene systems.

We consider a suspended monolayer graphene system as shown in Figure S1. The unique optical properties of graphene enable it to support two different modes of graphene plasmons, i.e., the transverse-electric (TE) graphene plasmon and transverse-magnetic (TM) graphene plasmon.

To determine dispersion surfaces of the TE and TM modes in the structure in Figure 1(a) of the main text, we establish the (h, y, ρ) coordinate system where the in-plane wavevector q of the eigenmode is parallel to the ρ-axis (Figure S2). The (h, y, ρ) coordinate system is transformed from the Cartesian (x, y, z) coordinate system in Figure 1(a) by

$$\begin{bmatrix} \hat{h} \\ \hat{\rho} \end{bmatrix} = \begin{bmatrix} \cos\theta & -\sin\theta \\ \sin\theta & \cos\theta \end{bmatrix} \begin{bmatrix} \hat{x} \\ \hat{z} \end{bmatrix} \tag{S1}$$

where $\tan\theta = k_x/k_z$; k_x and k_z are respectively the components of wavevector along the x and z directions. In this rotated coordinate system, the field components of TE mode (corresponding to the TE graphene plasmons) take the form of $\vec{E}_{\mathrm{TE}} = \hat{h}E_h$ and $\vec{H}_{\mathrm{TE}} = \hat{y}H_y + \hat{\rho}H_\rho$, while those of TM mode (corresponding to the TM graphene plasmons) are $\vec{E}_{\mathrm{TM}} = \hat{y}E_y + \hat{\rho}E_\rho$ and $\vec{H}_{\mathrm{TM}} = \hat{h}H_h$. The scattering fields of the TE and TM modes can be respectively expressed by Eqs. (S2) and (S3):

$$E_h = \begin{cases} \left(R_{s_{1'}} e^{\mathrm{i}k_{y1'}y}\right) e^{\mathrm{i}q\rho}, & 0 < y \\ \left(T_{s_{1''}} e^{-\mathrm{i}k_{y1''}y} + R_{s_{1''}} e^{\mathrm{i}k_{y1''}y}\right) e^{\mathrm{i}q\rho}, & -d < y < 0 \\ \left(T_{s_2} e^{-\mathrm{i}k_{y2}y}\right) e^{\mathrm{i}q\rho}, & y < -d \end{cases} \tag{S2a}$$

$$H_y = \begin{cases} \dfrac{q}{\omega\mu_0}\left(R_{s_{1'}} e^{\mathrm{i}k_{y1'}y}\right) e^{\mathrm{i}q\rho}, & 0 < y \\ \dfrac{q}{\omega\mu_0}\left(T_{s_{1''}} e^{-\mathrm{i}k_{y1''}y} + R_{s_{1''}} e^{\mathrm{i}k_{y1''}y}\right) e^{\mathrm{i}q\rho}, & -d < y < 0 \\ \dfrac{q}{\omega\mu_0}\left(T_{s_2} e^{-\mathrm{i}k_{y2}y}\right) e^{\mathrm{i}q\rho}, & y < -d \end{cases} \tag{S2b}$$

$$H_\rho = \begin{cases} -\dfrac{k_{y_{1'}}}{\omega\mu_0}\left(R_{s_{1'}} e^{\mathrm{i}k_{y_{1'}}y}\right)e^{\mathrm{i}q\rho}, & 0 < y \\ -\dfrac{k_{y_{1''}}}{\omega\mu_0}\left(-T_{s_{1''}} e^{-\mathrm{i}k_{y_{1''}}y} + R_{s_{1''}} e^{\mathrm{i}k_{y_{1''}}y}\right)e^{\mathrm{i}q\rho}, & -d < y < 0 \\ \dfrac{k_{y_2}}{\omega\mu_0}\left(T_{s_2} e^{-\mathrm{i}k_{y_2}y}\right)e^{\mathrm{i}q\rho}, & y < -d \end{cases} \qquad \text{(S2c)}$$

$$H_h = \begin{cases} -\omega\varepsilon_0\varepsilon_{1'}\left(R_{p_{1'}} e^{\mathrm{i}k_{y_{1'}}y}\right)e^{\mathrm{i}q\rho}, & 0 < y \\ -\omega\varepsilon_0\varepsilon_{1'}\left(T_{p_{1''}} e^{-\mathrm{i}k_{y_{1''}}y} + R_{p_{1''}} e^{\mathrm{i}k_{y_{1''}}y}\right)e^{\mathrm{i}q\rho}, & -d < y < 0 \\ -\omega\varepsilon_0\varepsilon_{1'}\left(T_{p_2} e^{-\mathrm{i}k_{y_2}y}\right)e^{\mathrm{i}q\rho}, & y < -d \end{cases} \qquad \text{(S3a)}$$

$$E_y = \begin{cases} q\left(R_{p_{1'}} e^{\mathrm{i}k_{y_{1'}}y}\right)e^{\mathrm{i}q\rho}, & 0 < y \\ q\dfrac{\varepsilon_{1'}}{\varepsilon_{1''}}\left(T_{p_{1''}} e^{-\mathrm{i}k_{y_{1''}}y} + R_{p_{1''}} e^{\mathrm{i}k_{y_{1''}}y}\right)e^{\mathrm{i}q\rho}, & -d < y < 0 \\ q\dfrac{\varepsilon_{1'}}{\varepsilon_2}\left(T_{p_2} e^{-\mathrm{i}k_{y_2}y}\right)e^{\mathrm{i}q\rho}, & y < -d \end{cases} \qquad \text{(S3b)}$$

$$E_\rho = \begin{cases} -k_{y_{1'}}\left(R_{p_{1'}} e^{\mathrm{i}k_{y_{1'}}y}\right)e^{\mathrm{i}q\rho}, & 0 < y \\ -k_{y_{1''}}\dfrac{\varepsilon_{1'}}{\varepsilon_{1''}}\left(-T_{p_{1''}} e^{-\mathrm{i}k_{y_{1''}}y} + R_{p_{1''}} e^{\mathrm{i}k_{y_{1''}}y}\right)e^{\mathrm{i}q\rho}, & -d < y < 0 \\ k_{y_2}\dfrac{\varepsilon_{1'}}{\varepsilon_2}\left(T_{p_2} e^{-\mathrm{i}k_{y_2}y}\right)e^{\mathrm{i}q\rho}, & y < -d \end{cases} \qquad \text{(S3c)}$$

where $k_{y_j} = \sqrt{\varepsilon_j k_0^2 - q^2}$ is the y component of wavevector in the region j, ε_0 and μ_0 are respectively the permittivity and permeability in free space, ω is the angular frequency, $k_0 = \omega/c$, $q = \sqrt{k_x^2 + k_z^2}$, $\rho = \sqrt{x^2 + z^2}$, $R_{s_j}\,(T_{s_j})$ and $R_{p_j}\,(T_{p_j})$ are the reflection (transmission) coefficients for the TE and TM modes in the region j, respectively. By enforcing the electromagnetic boundary conditions at $y = 0$ and $y = -d$, the dispersion relations of graphene plasmons for the TE and TM modes are given by Eqs. (S4) and (S5), respectively, which read:

$$\left(\frac{k_{y_{1''}} + k_{y_{1'}} + \sigma_{\mathrm{g}}\omega\mu_0}{k_{y_{1''}} - k_{y_{1'}} - \sigma_{\mathrm{g}}\omega\mu_0}\right)\left(\frac{k_{y_{1''}} + k_{y_2}}{k_{y_{1''}} - k_{y_2}}\right) = e^{2\mathrm{i}k_{y_{1''}}d} \qquad \text{(S4)}$$

$$-\frac{\left(\frac{\varepsilon_{1'}}{k_{y_{1'}}}+\frac{\varepsilon_{1''}}{k_{y_{1''}}}+\frac{\sigma_{\mathrm{g}}}{\omega\varepsilon_0}\right)\left(\frac{\varepsilon_{1''}}{k_{y_{1''}}}+\frac{\varepsilon_2}{k_{y_2}}\right)}{\left(\frac{\varepsilon_{1'}}{k_{y_{1'}}}-\frac{\varepsilon_{1''}}{k_{y_{1''}}}+\frac{\sigma_{\mathrm{g}}}{\omega\varepsilon_0}\right)\left(\frac{\varepsilon_{1''}}{k_{y_{1''}}}-\frac{\varepsilon_2}{k_{y_2}}\right)}=e^{2\mathrm{i}k_{y_{1''}}d} \tag{S5}$$

For the suspended graphene structure in Figure S1, Eqs. (S4) and (S5) can be further simplified through the identities $\varepsilon_1=\varepsilon_{1'}=\varepsilon_{1''}$ and $k_{y_1}=k_{y_{1'}}=k_{y_{1''}}$ as

$$\left(\frac{2k_{y_1}+\sigma_{\mathrm{g}}\omega\mu_0}{-\sigma_{\mathrm{g}}\omega\mu_0}\right)\left(\frac{k_{y_1}+k_{y_2}}{k_{y_1}-k_{y_2}}\right)=e^{2\mathrm{i}k_{y_1}d} \tag{S6}$$

$$\frac{\left(2\frac{\varepsilon_1}{k_{y_1}}+\frac{\sigma_{\mathrm{g}}}{\omega\varepsilon_0}\right)\left(\frac{\varepsilon_1}{k_{y_1}}+\frac{\varepsilon_2}{k_{y_2}}\right)}{\left(\frac{\sigma_{\mathrm{g}}}{\omega\varepsilon_0}\right)\left(\frac{\varepsilon_1}{k_{y_1}}-\frac{\varepsilon_2}{k_{y_2}}\right)}=e^{2\mathrm{i}k_{y_1}d} \tag{S7}$$

Here, σ_{g} denotes the conductivity of graphene, which is described by the Kubo formula as [1]:

$$\sigma_{\mathrm{g}}(\omega)=\frac{e^2\mu_{\mathrm{c}}}{\pi\hbar^2}\frac{\mathrm{i}}{\omega+\mathrm{i}\tau^{-1}}+\frac{e^2}{4\hbar}\left[\Theta(\hbar\omega-2\mu_{\mathrm{c}})+\frac{\mathrm{i}}{\pi}\log\left|\frac{\hbar\omega-2\mu_{\mathrm{c}}}{\hbar\omega+2\mu_{\mathrm{c}}}\right|\right] \tag{S8}$$

where μ_{c} and τ are respectively the chemical potential and relaxation time in graphene, $\hbar$ is the reduced Planck constant, and Θ is the Heaviside step function.

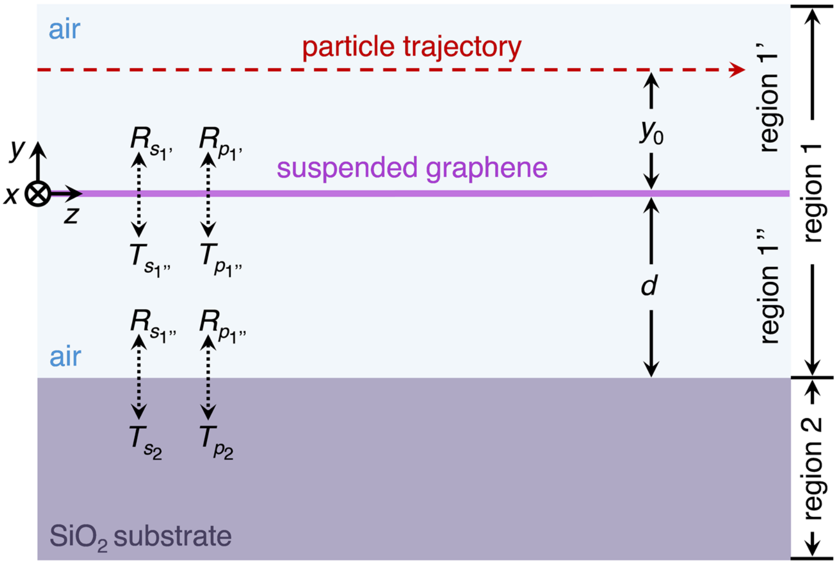

Figure S1 | Schematic of the suspended graphene layered structure. The permittivity of region

j is denoted by ε_j. The suspended graphene divides region 1 into region 1' and region 1'', that is $\varepsilon_1 = \varepsilon_{1'} = \varepsilon_{1''}$. Region 2 (silicon dioxide, SiO_2) with ε_2 serves as the substrate. The thickness of the suspended layer (region 1'') is d. The charged particle travels in region 1' along the z-axis with the particle-graphene separation y_0. R_{sj} (T_{sj}) and R_{pj} (T_{pj}) are the reflection (transmission) coefficients for TE- and TM- polarized light in the region j, respectively.

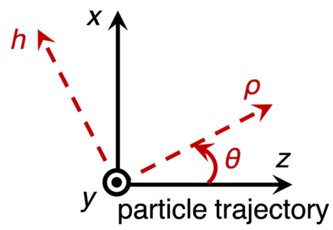

Figure S2 | Creation of Cartesian coordinate systems for the charged particle in the suspended graphene structure. The particle trajectory aligns with the z-axis in (x, y, z) coordinate system. The in-plane wavevector q aligns with the ρ-axis in (h, y, ρ) coordinate system. Here the (h, y, ρ) coordinate system is derived by rotating the (x, y, z) coordinate system around the y-axis by an angle θ, where the y-axis remains the out-of-plane direction in both coordinate systems.

Section S2. Calculation of the radiation pattern of graphene plasmon Cherenkov radiation.

Within the framework of classical electrodynamics [2], the current density induced by the charged particle with a velocity of $\overline{v}_e = \hat{z} v_e$ in the (x, y, z) coordinate system as shown in Figure 1a is

$$\overline{J}\left(\overline{r},t\right) = \hat{z} v_e e \delta\left(x\right) \delta\left(y - y_0\right) \delta\left(z - v_e t\right) \tag{S9}$$

where e is the elementary charge. The vector potential of the system is formulated as $\overline{H} = \frac{1}{\mu_0} \nabla \times \overline{\phi}_0$.

The current density that is polarized solely in the z-direction, inducing vector potential with the

same polarization, i.e., $\bar{\phi}_0 = \hat{z}\phi_0$, that is given by

$$\bar{\phi}_0 = \hat{z}\phi_0 = \hat{z}\left(\frac{\mathrm{i}e}{8\pi}H_0^{(1)}\left(q\rho\right)e^{\mathrm{i}k_z z}\right) \tag{S10}$$

where $H_0^{(1)}$ is the zeroth order Hankel function of the first kind and $k_z = \omega / v_\mathrm{e}$. In (*x*, *y*, *z*) coordinate system, Eq. (S10) can be expanded to [3]

$$\bar{\phi}_0 = \hat{z}\int_{-\infty}^{+\infty}\mathrm{d}k_x\left(\frac{\mathrm{i}e}{8\pi^2 k_{y_1}}\right)e^{\mathrm{i}k_x x+\mathrm{i}k_{y_1}\left|y-y_0\right|+\mathrm{i}k_z z} \tag{S11}$$

Correspondingly, the source field in frequency domain can be expressed as:

$$\begin{cases}\bar{E}\left(\bar{r},\omega\right)=\dfrac{\mathrm{i}}{\omega\varepsilon_0\varepsilon_1}\nabla\times\nabla\times\bar{\phi}_0\\ \bar{H}\left(\bar{r},\omega\right)=\nabla\times\bar{\phi}_0\end{cases} \tag{S12}$$

Therefore, the field components of the swift charged particle source in (*x*, *y*, *z*) coordinate system are given by

$$E_x^s\left(\bar{r},\omega\right)=\frac{1}{\omega\varepsilon_0\varepsilon_1}\int\mathrm{d}k_x\left(\frac{e}{8\pi^2 k_{y_1}}\right)\left(k_x k_z\right)e^{\mathrm{i}k_x x+\mathrm{i}k_{y_1}\left|y-y_0\right|+\mathrm{i}k_z z}$$

$$E_y^s\left(\bar{r},\omega\right)=\frac{1}{\omega\varepsilon_0\varepsilon_1}\int\mathrm{d}k_x\left(\frac{e}{8\pi^2 k_{y_1}}\right)\left(\mathrm{sgn}\left(y-y_0\right)k_{y_1}k_z\right)e^{\mathrm{i}k_x x+\mathrm{i}k_{y_1}\left|y-y_0\right|+\mathrm{i}k_z z}$$

$$E_z^s\left(\bar{r},\omega\right)=\frac{1}{\omega\varepsilon_0\varepsilon_1}\int\mathrm{d}k_x\left(\frac{e}{8\pi^2 k_{y_1}}\right)\left(-\left(k_x^2+k_{y_1}^2\right)\right)e^{\mathrm{i}k_x x+\mathrm{i}k_{y_1}\left|y-y_0\right|+\mathrm{i}k_z z} \tag{S13}$$

$$H_x^s\left(\bar{r},\omega\right)=\int\mathrm{d}k_x\left(\frac{e}{8\pi^2 k_{y_1}}\right)\left(-\mathrm{sgn}\left(y-y_0\right)k_{y_1}\right)e^{\mathrm{i}k_x x+\mathrm{i}k_{y_1}\left|y-y_0\right|+\mathrm{i}k_z z}$$

$$H_y^s\left(\bar{r},\omega\right)=\int\mathrm{d}k_x\left(\frac{e}{8\pi^2 k_{y_1}}\right)\left(k_x\right)e^{\mathrm{i}k_x x+\mathrm{i}k_{y_1}\left|y-y_0\right|+\mathrm{i}k_z z}$$

$$H_z^s\left(\bar{r},\omega\right)=0$$

For the convenience of subsequent analysis and discussion, we use the aforementioned rotation matrix in Eq. (S1) to transform each source field components in (*x*, *y*, *z*) coordinate system to ones in (*h*, *y*, *ρ*) coordinate system, where the *y*-direction field component remains invariant. For the (*x*,

y, z) coordinate system in Figure 1, only the TE-polarized component contains the component $H_y^s\left(\bar{r},\omega\right)$, and only the TM-polarized component contains the component $E_y^s\left(\bar{r},\omega\right)$. As such, the field components of $H_y^s\left(\bar{r},\omega\right)$ and $E_y^s\left(\bar{r},\omega\right)$ can be used to determine the TE- and TM-polarized components of source field in (h, y, ρ) coordinate system, respectively. By applying Gauss's law ($\nabla\cdot\bar{E}=0$) and Faraday's law ($\nabla\times\bar{E}=\mathrm{i}\omega\mu_0\bar{H}$), the TE-polarized components can be calculated as

$$E_h^s=\left(\frac{e}{8\pi^2k_{y_1}}\right)\left(\frac{1}{\omega\varepsilon_0\varepsilon_1}\right)\left(\frac{\varepsilon_1k_xk_0^2}{q}\right)e^{ik_{y_1}|y-y_0|}e^{\mathrm{i}q\rho}$$

$$H_y^s=\left(\frac{e}{8\pi^2k_{y_1}}\right)\left(k_x\right)e^{\mathrm{i}k_{y_1}|y-y_0|}e^{\mathrm{i}q\rho} \tag{S14}$$

$$H_\rho^s=\left(\frac{e}{8\pi^2k_{y_1}}\right)\left(-\frac{k_xk_{y_1}}{q}\right)\mathrm{sgn}\left(y-y_0\right)e^{\mathrm{i}k_{y_1}|y-y_0|}e^{\mathrm{i}q\rho}$$

and the TM-polarized components can be calculated as

$$H_h^s=\left(\frac{e}{8\pi^2k_{y_1}}\right)\left(-\frac{k_{y_1}k_z}{q}\right)\mathrm{sgn}\left(y-y_0\right)e^{\mathrm{i}k_{y_1}|y-y_0|}e^{\mathrm{i}q\rho}$$

$$E_y^s=\left(\frac{e}{8\pi^2k_{y_1}}\right)\left(\frac{1}{\omega\varepsilon_0\varepsilon_1}\right)\left(k_{y_1}k_z\right)\mathrm{sgn}\left(y-y_0\right)e^{\mathrm{i}k_{y_1}|y-y_0|}e^{\mathrm{i}q\rho} \tag{S15}$$

$$E_\rho^s=\left(\frac{e}{8\pi^2k_{y_1}}\right)\left(\frac{1}{\omega\varepsilon_0\varepsilon_1}\right)\left(-\frac{k_{y_1}^2k_z}{q}\right)e^{\mathrm{i}k_{y_1}|y-y_0|}e^{\mathrm{i}q\rho}$$

Now the total radiation fields are expressed as the sum of the source field and the scattering field in the suspended graphene structure. Via matching the boundary conditions, we obtain the following expressions:

1) the scattering coefficients for the TE mode are

$$R_{s_{1'}}=\frac{a_1-a_2}{s_1+s_2}$$

$$T_{s_{1''}}=\frac{a_3}{s_1+s_2} \tag{S16}$$

$$R_{s_{1''}} = \frac{a_4}{s_1 + s_2}$$

$$T_{s_2} = \frac{-a_5}{s_1 + s_2}$$

where

$$s_1 = \left(2k_{y_{1''}} + \omega\mu_0\sigma_{\mathrm{g}}\right)\left(k_{y_{1''}} + k_{y_2}\right)e^{-\mathrm{i}k_{y_{1''}}d}$$

$$s_2 = \omega\mu_0\sigma_{\mathrm{g}}\left(k_{y_{1''}} - k_{y_2}\right)e^{\mathrm{i}k_{y_{1''}}d}$$

$$a_1 = \left(2k_{y_{1''}} - \omega\mu_0\sigma_{\mathrm{g}}\right)\left(k_{y_{1''}} - k_{y_2}\right)e^{\mathrm{i}k_{y_{1''}}d}$$

$$a_2 = \omega\mu_0\sigma_{\mathrm{g}}\left(k_{y_{1''}} + k_{y_2}\right)e^{-\mathrm{i}k_{y_{1''}}d} \tag{S17}$$

$$a_3 = 2k_{y_{1''}}\left(k_{y_{1''}} + k_{y_2}\right)e^{-\mathrm{i}k_{y_{1''}}d}$$

$$a_4 = 2k_{y_{1''}}\left(k_{y_{1''}} - k_{y_2}\right)e^{\mathrm{i}k_{y_{1''}}d}$$

$$a_5 = 4k_{y_{1''}}k_{y_2}e^{-\mathrm{i}k_{y_{1''}}d}$$

2) the scattering coefficients for the TM mode are

$$R_{p_{1'}} = \frac{b_1 - b_2}{p_1 + p_2}$$

$$T_{p_{1''}} = \frac{b_3}{p_1 + p_2} \tag{S18}$$

$$R_{p_{1''}} = \frac{b_4}{p_1 + p_2}$$

$$T_{p_2} = \frac{b_5}{p_1 + p_2}$$

where

$$p_1 = \left(2\frac{k_{y_{1''}}}{\varepsilon_{1''}} + \frac{k_{y_{1''}}^2\sigma_{\mathrm{g}}}{\omega\varepsilon_0\varepsilon_{1''}^2}\right)\left(\frac{k_{y_{1''}}}{\varepsilon_{1''}} + \frac{k_{y_2}}{\varepsilon_2}\right)e^{-\mathrm{i}k_{y_{1''}}d}$$

$$p_2 = \frac{k_{y_{1''}}^2\sigma_{\mathrm{g}}}{\omega\varepsilon_0\varepsilon_{1''}^2}\left(\frac{k_{y_{1''}}}{\varepsilon_{1''}} - \frac{k_{y_2}}{\varepsilon_2}\right)e^{\mathrm{i}k_{y_{1''}}d}$$

$$b_1 = \left(2\frac{k_{y_{1''}}}{\varepsilon_{1''}} - \frac{k_{y_{1''}}^2\sigma_{\mathrm{g}}}{\omega\varepsilon_0\varepsilon_{1''}^2}\right)\left(\frac{k_{y_{1''}}}{\varepsilon_{1''}} - \frac{k_{y_2}}{\varepsilon_2}\right)e^{\mathrm{i}k_{y_{1''}}d}$$

$$b_2 = \frac{k_{y_{1''}}^2\sigma_{\mathrm{g}}}{\omega\varepsilon_0\varepsilon_{1''}^2}\left(\frac{k_{y_{1''}}}{\varepsilon_{1''}} + \frac{k_{y_2}}{\varepsilon_2}\right)e^{-\mathrm{i}k_{y_{1''}}d} \tag{S19}$$

$$b_3 = 2\frac{k_{y_{1''}}}{\varepsilon_{1''}}\left(\frac{k_{y_{1''}}}{\varepsilon_{1''}} + \frac{k_{y_2}}{\varepsilon_2}\right)e^{-\mathrm{i}k_{y_{1''}}d}$$

$$b_4 = 2\frac{k_{y_{1''}}}{\varepsilon_{1''}}\left(\frac{k_{y_{1''}}}{\varepsilon_{1''}} - \frac{k_{y_2}}{\varepsilon_2}\right)e^{\mathrm{i}k_{y_{1''}}d}$$

$$b_5 = 4\frac{k_{y_{1''}}}{\varepsilon_{1''}}\frac{k_{y_2}}{\varepsilon_2}e^{-\mathrm{i}k_{y_{1''}}d}$$

After the above calculation, we can obtain the expression of the radiation pattern of Cherenkov radiation by applying the reversed rotation matrix on the field components. For example, the TE-polarized field distribution $E_{\mathrm{TE},z}$ is determined as

$$E_{\mathrm{TE},z}(x,z) = \frac{1}{\omega\varepsilon_0\varepsilon_1}\int_{+\infty}^{-\infty}\mathrm{d}k_x\left[\left(A_{s_0} + A_{s_0}R_{\mathrm{TE}}\right)\right]e^{\mathrm{i}k_x x+\mathrm{i}k_{y_1}|y-y_0|+\mathrm{i}k_z z} \tag{S20}$$

where $A_{s_0} = \frac{e}{8\pi^2}\frac{1}{k_{y_1}}\sin^2\theta$ is the amplitude of TE-polarized incident field and the reflection coefficient R_{TE} corresponds to the $R_{s1'}$ in Eq. (S16). Particularly, at $y = 0$, we have

$$E_{\mathrm{TE},z}(x,z)\big|_{y=0} = \mathrm{i}\frac{e\omega^2\mu_0}{8\pi^2}\int_{-\pi/2}^{\pi/2}\mathrm{d}\theta\frac{1}{\cos\theta|\cos\theta|}\left[\left(\frac{1}{k_{y_1}}\sin^2\theta(1+R_{\mathrm{TE}})\right)e^{\mathrm{i}k_{y_1}y_0}\right]e^{\mathrm{i}\left(\frac{\omega}{v_\mathrm{e}}\tan\theta\right)x+\mathrm{i}\left(\frac{\omega}{v_\mathrm{e}}\right)z} \tag{S21}$$

And the TM-polarized field distribution $E_{\mathrm{TM},z}$ is determined as

$$E_{\mathrm{TM},z}(x,z) = \frac{1}{\omega\varepsilon_0\varepsilon_1}\int_{+\infty}^{-\infty}\mathrm{d}k_x\left[\left(A_{p_0} + A_{p_0}R_{\mathrm{TM}}\right)\right]e^{\mathrm{i}k_x x+\mathrm{i}k_{y_1}|y-y_0|+\mathrm{i}k_z z} \tag{S22}$$

where $A_{p_0} = \frac{e}{8\pi^2}\frac{k_{y_1}}{k^2}\cos^2\theta$ is the amplitude of TM-polarized incident field and the reflection coefficient R_{TM} corresponds to the $R_{p1'}$ in Eq. (S18). Particularly, at $y = 0$, we have

$$E_{\mathrm{TM},z}(x,z)\big|_{y=0} = \mathrm{i}\frac{e\omega^2\mu_0}{8\pi^2}\int_{-\pi/2}^{\pi/2}\mathrm{d}\theta\frac{1}{\cos\theta|\cos\theta|}\left[\left(\frac{k_{y_1}}{k^2}\cos^2\theta(1+R_{\mathrm{TM}})\right)e^{\mathrm{i}k_{y_1}y_0}\right]e^{\mathrm{i}\left(\frac{\omega}{v_\mathrm{e}}\tan\theta\right)x+\mathrm{i}\left(\frac{\omega}{v_\mathrm{e}}\right)z} \tag{S23}$$

where $k = \sqrt{\varepsilon_1}k_0$.

<u>Section S3. Calculation of the angular spectrum of graphene plasmon Cherenkov radiation.</u>

Regarding the response to the moving charged particle, since the particle moves only along the

z-direction, the energy loss of Cherenkov radiation can be calculated by using only the zz component of induced dyadic Green's function $G_{zz,\text{ind}}\left(\bar{r},\bar{r}'\right)$ as [4,5,6]

$$\Gamma(\omega) = -\frac{4\alpha}{c}\int \mathrm{d}z \int \mathrm{d}z' \,\mathrm{Im}\left[G_{zz,\text{ind}}\left(\bar{r},\bar{r}'\right) e^{-\mathrm{i}\frac{\omega}{v_\mathrm{e}}(z-z')} \right] \tag{S24}$$

where $\alpha = \dfrac{e^2}{4\pi\varepsilon_0 \hbar c}$ is the fine structure constant. The $G_{zz,\text{ind}}\left(\bar{r},\bar{r}'\right)$ is given by

$$G_{zz,\text{ind}}\left(\bar{r},\bar{r}'\right) = \frac{1}{8\pi^2}\int_0^\infty q\mathrm{d}q \int_0^{2\pi} \mathrm{d}\theta \frac{1}{k_{y_1}}\left[\sin^2\theta R_\mathrm{TE} e^{\mathrm{i}k_{y_1}y_0} + \frac{k_{y_1}^2}{k^2}\cos^2\theta R_\mathrm{TM} e^{\mathrm{i}k_{y_1}y_0} \right] e^{\mathrm{i}q\sin\theta x + \mathrm{i}q\cos\theta z + \mathrm{i}k_{y_1}y} \tag{S25}$$

Below we first investigate the energy loss induced by TE graphene plasmon Cherenkov radiation in the suspended graphene structure. Since the TE-polarized component of energy loss is related to the factor containing reflection coefficient R_TE in Eq. (S25), its expression can be written as

$$\Gamma_\mathrm{TE}(\omega) = \frac{2\alpha L}{\pi c}\mathrm{Re}\int_0^\infty q\mathrm{d}q \int_0^{\frac{\pi}{2}} \mathrm{d}\theta \left[\frac{1}{k_{y_1}} \sin^2\theta R_\mathrm{TE} e^{2\mathrm{i}k_{y_1}y_0} \delta\left(q\cos\theta - \frac{\omega}{v_\mathrm{e}} \right) \right] \tag{S26}$$

where L is the interaction length of the charged particle. By using the identity $\delta\left(q\cos\theta - \dfrac{\omega}{v_\mathrm{e}} \right) = \dfrac{1}{q}\delta\left(\cos\theta - \dfrac{\omega}{q v_\mathrm{e}} \right)$, Eq. (S26) can be simplified as

$$\Gamma_\mathrm{TE}(\omega) = \frac{2\alpha L}{\pi c}\mathrm{Re}\int_{\omega/v_\mathrm{e}}^\infty \mathrm{d}q \left[\frac{|\sin\theta|}{k_{y_1}} R_\mathrm{TE} e^{2\mathrm{i}k_{y_1}y_0} \right] \tag{S27}$$

Thus, the TE-polarized component of the power emitted by the charged particle can be derived from the energy loss $\Gamma_\mathrm{TE}(\omega)$ as

$$\begin{aligned} P_\mathrm{TE} &= \int_0^\infty \mathrm{d}\omega \Gamma_\mathrm{TE}(\omega) \hbar\omega \frac{v_\mathrm{e}}{L} \\ &= \frac{e^2 \mu_0 \omega v_\mathrm{e}}{2\pi^3} \int_0^\infty \mathrm{d}\omega \int_{\omega/v_\mathrm{e}}^\infty \mathrm{d}q \left[\frac{|\sin\theta|}{k_{y_1}} R_\mathrm{TE} e^{2\mathrm{i}k_{y_1}y_0} \right] \end{aligned} \tag{S28}$$

Through similar derivations, the TM-polarized component of energy loss can be written as

$$\Gamma_{\mathrm{TM}}(\omega)=\frac{2\alpha L}{\pi c}\mathrm{Re}\int_0^\infty q\mathrm{d}q\int_0^{\frac{\pi}{2}}\mathrm{d}\theta\left[\frac{k_{y_1}}{k^2}\cos^2\theta R_{\mathrm{TM}}e^{2\mathrm{i}k_{y_1}y_0}\delta\left(q\cos\theta-\frac{\omega}{v_{\mathrm{e}}}\right)\right] \tag{S29}$$

Thus, the TM-polarized component of the power emitted by the charged particle can be derived from the energy loss $\Gamma_{\mathrm{TM}}(\omega)$ as

$$\begin{aligned} P_{\mathrm{TM}} &= \int_0^\infty \mathrm{d}\omega\Gamma_{\mathrm{TM}}(\omega)\hbar\omega\frac{v_{\mathrm{e}}}{L} \\ &= \frac{e^2\mu_0\omega v_{\mathrm{e}}}{2\pi^3}\int_0^\infty \mathrm{d}\omega\int_{\omega/v_{\mathrm{e}}}^\infty \mathrm{d}q\left[\frac{k_{y_1}\cos^2\theta}{k^2\left|\sin\theta\right|}R_{\mathrm{TM}}e^{2\mathrm{i}k_{y_1}y_0}\right] \end{aligned} \tag{S30}$$

Section S4. Influence of host materials on detectable momentum of Cherenkov detectors.

To clarify why the momentum detection range is limited, we revisit the mechanism of Cherenkov detectors. Particles with different masses will have different particle velocities at a fixed momentum, as $p=m_0v_{\mathrm{e}}\left[1-\left(v_{\mathrm{e}}/c\right)^2\right]^{-\frac{1}{2}}$, where p is the particle momentum and m_0 is the particle rest mass. Due to different particle velocities, these particles would emit photons at distinct Cherenkov angles satisfying $\cos\theta=c/nv_{\mathrm{e}}$. In other words, by measuring the Cherenkov angles, one can discriminate different particles. On the other hand, the performance of Cherenkov detectors relies on the sensitivity of Cherenkov angle to particle velocity. In general, high-sensitivity detection of relativistic particles requires their velocities (or momenta) to be sufficiently close to the threshold, i.e., $v_{\mathrm{e}}\rightarrow v_{\mathrm{th}}=c/n$ (Figure S3). Thus, if the refractive index of host material is not close enough to unity, the small velocity (or momentum) threshold prevents the Cherenkov detector from particle detection in high momentum regime. Specifically, a charged particle must travel faster than the Cherenkov threshold velocity to emit photons, and the number of emitted photons increases with their velocity. However, when the velocity (and consequently the momentum) becomes excessively high, the emission angles of the photons converge to a maximum value that is identical for all

particle types in a given material (corresponding to the converging region of the curves in Figure 4). As a result, the photon yield also becomes indistinguishable among different particle species. In this regime, it is no longer possible to identify particle types based on either the emission angle or the photon count.

For example, for traditional silica aerogel detectors with a refractive index n in the range of 1.005 to 1.060 (with corresponding v_{th} limited to the range from 0.943c to 0.995c), the working momentum can only be within 10 GeV/c to discriminate four elementary particles (electron, pion, kaon, and proton, with rest masses of 0.511 MeV/c^2, 139.6 MeV/c^2, 493.7 MeV/c^2, and 938.3 MeV/c^2, respectively). As another example, quartz has a refractive index n around 1.4 (with v_{th} as small as 0.714c), and its corresponding momentum coverage for the identification of pions and kaons is typically limited to be below 6 GeV/c [7,8].

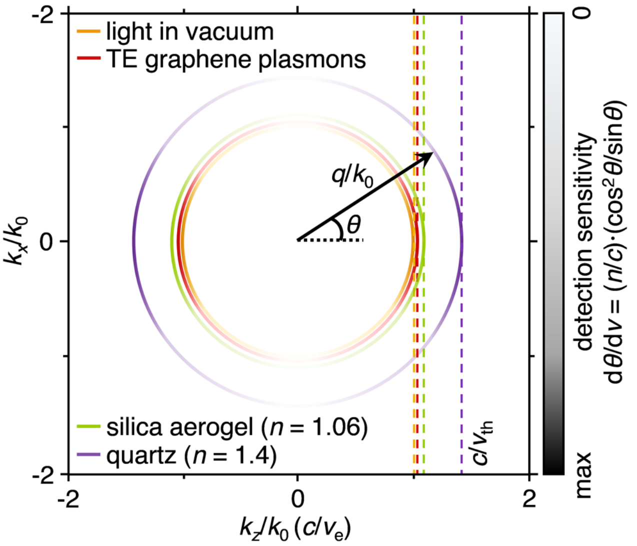

Figure S3 | The influence of particle velocity on detection sensitivity under different media. The circular curves represent the isofrequency contours of four media (vacuum, TE graphene plasmons, silica aerogel, and quartz), where the detection sensitivity is characterized by the depth of color. The dashed lines correspond to the cutoff wavevectors of Cherenkov radiation induced by

velocity thresholds.

Section S5. Radiation field pattern of TE graphene plasmon Cherenkov radiation.

The field pattern of TE graphene plasmon Cherenkov radiation is sensitive to relativistic particle velocity. To illustrate this point, Figure S4 plots the field distributions of Cherenkov radiation produced by a charged particle with velocity higher than the velocity threshold. If the normalized particle velocity is β = 0.9999, 0.9994, and 0.9989, the emission angle is θ = 2.808°, 2.144°, and 1.146°, respectively. Here, $\beta = v_e/c$ is the particle velocity normalized by the light speed in vacuum.

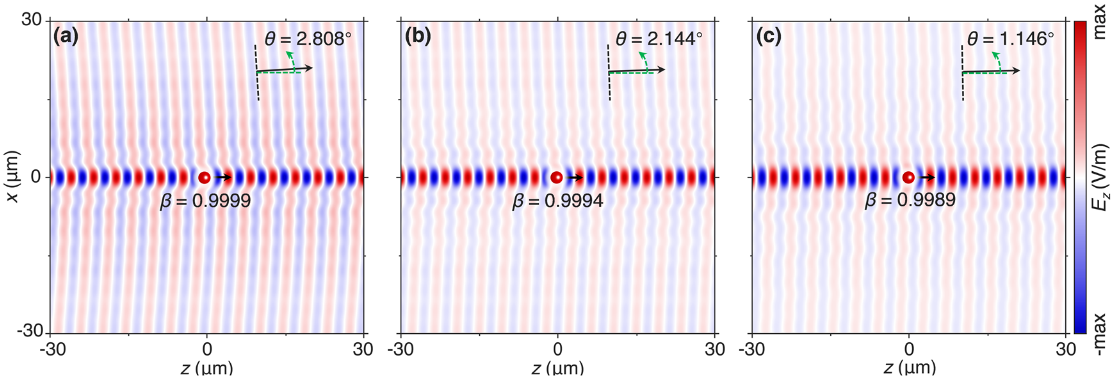

Figure S4 | Field distributions of TE graphene plasmon Cherenkov radiation. (a-c) Field distributions of TE graphene plasmon Cherenkov radiation. The studied particle velocities are β = 0.9999, 0.9994 and 0.9989, respectively, leading to the emission angle of 2.808°, 2.144° and 1.146°, respectively. Here, the particle velocity is normalized by the light speed in vacuum, i.e., $\beta = v_e/c$.

Section S6. More discussions on the influence of relaxation time on the radiation behaviors.

In this section, we discuss the influence of the relaxation time on the angular line width and

the angular power spectral density of TE graphene plasmon Cherenkov radiation. Here, the angular line width is defined as full width at half maxima of the angular power spectral density versus the Cherenkov angle, denoted as $\Delta\theta$. Our results indicate that there is a sharp energy enhancement only when the radiation behavior satisfies the equation $\cos\theta = \omega/(qv_e)$, i.e., the excitation of TE graphene plasmons. From Figure S5, extending (shortening) the relaxation time τ makes such energy enhancements become sharper (gentler). Taking $\beta = 0.9994$ as an example, when τ increases from 0.03 ps to 0.12 ps, the angular line width $\Delta\theta$ reduces from 0.25° to 0.08°, which enhances the directionality of Cherenkov radiation. Moreover, the maximum angular power spectral density exhibits a linear increase with the relaxation time, as shown by the colorbars in Figure S5.

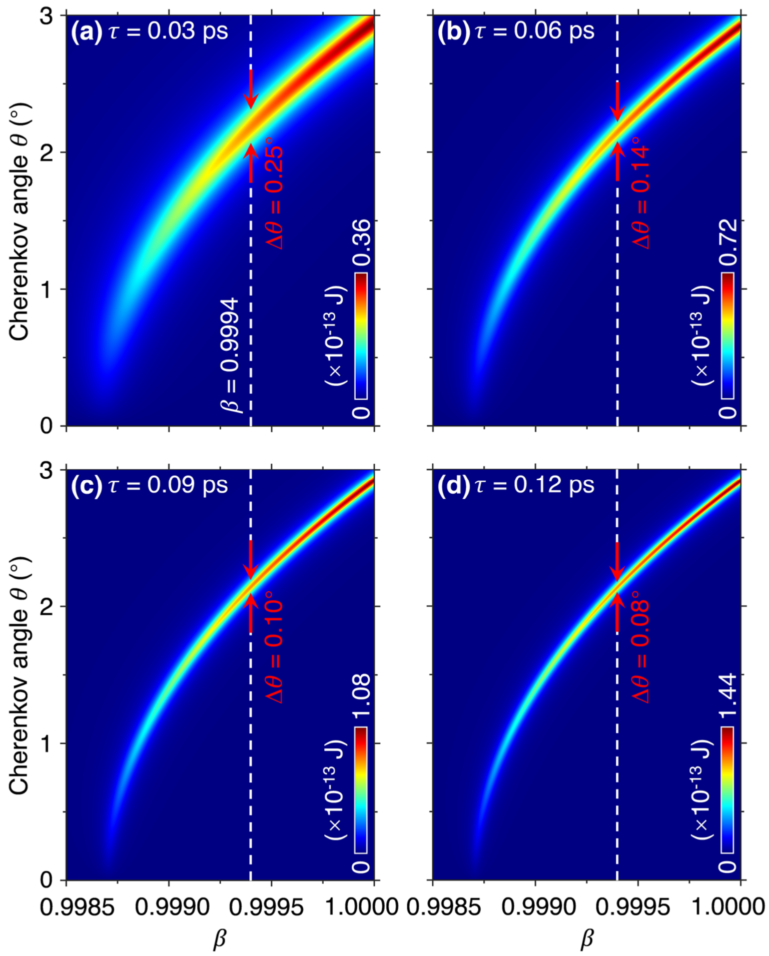

Figure S5 | The angular power spectral density as a function of the normalized particle velocity

***β* and the Cherenkov angle *θ* under different values of relaxation time *τ*.** In all the panels, the red marks indicate the angular line width, with the studied particle velocity $\beta = 0.9994$ marked by white dashed lines.

Section S7. More discussions on the influence of chemical potential on the performance of particle detection.

To further analyze the influence of chemical potential on the effective detection momentum range, we plot the relation between the particle velocity and the Cherenkov angle at different chemical potential values in Figure S6. We can see that the Cherenkov angle θ is more sensitive to the particle velocity v_e as v_e approaches Cherenkov threshold v_{th}. The Cherenkov threshold increases with increasing chemical potential. In principle, this study can enable high-sensitivity particle identification within any desired momentum range by adjusting the chemical potential μ_c via modulation of the external gate voltage.

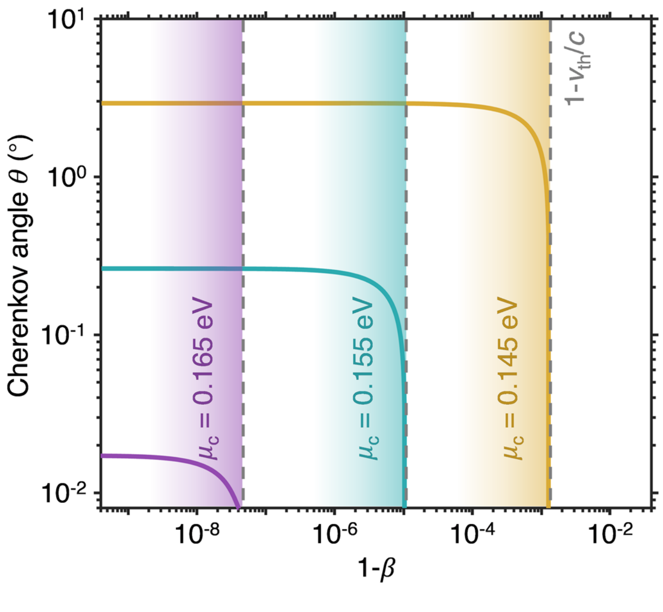

Figure S6 | Cherenkov angle *θ* as a function of 1-*β* under different values of chemical potential

$\boldsymbol{\mu}_\mathbf{c}$**.** Here $\beta = v_e/c$ is the normalized particle velocity. The shaded region indicates high sensitivity in particle detection, where the Cherenkov angle θ changes sharply as 1-β varies.

In realistic scenario, graphene samples typically exhibit structural defects and atomic contaminants that may affect their refractive index. To investigate the impact of these imperfections on the performance of proposed detectors, we plot in Figure S7 the spectral energy density as a function of emission angle for three charged particles to be measured. These results reflect that our platform remains capable of particle detection under moderate material imperfections. In the case where the chemical potential μ_c is initially set as 0.155 eV, three particles are separatable in the angular spectrum. To simulate the material imperfections, variations $\Delta\mu_c$ are added to the above chemical potential. When $\Delta\mu_c$ = -0.004 eV, the three particles can still be separated. However, if $\Delta\mu_c$ $\leq$ -0.006 eV, distinguishing between kaons and pions becomes difficult.

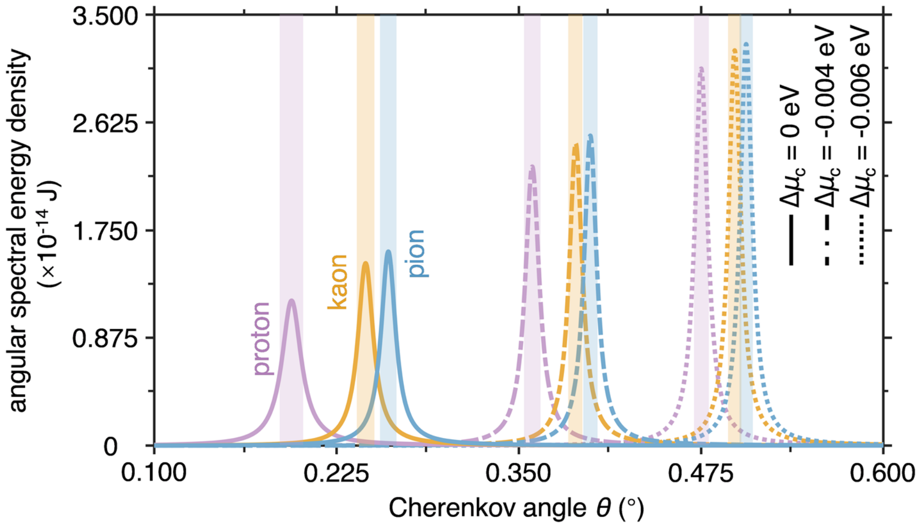

Figure S7 | Influence of the chemical potential variation $\boldsymbol{\Delta\mu_c}$ on the particle detection performance when the initial chemical potential $\boldsymbol{\mu_c}$ is set to 0.155 eV. Here, the particle momenta

are fixed at 300 GeV/*c*. The full width at half maxima of the angular power spectral density highlighted in the shaded region is used to qualify the angular resolution of particle detection.

Section S8. More discussions on the influence of particle-graphene separation on the radiation intensity.

To illustrate that TE graphene plasmons still maintain high emission efficiency at a separation distance on the order of micrometers, we present how the emission intensities of TE and TM graphene plasmon Cherenkov radiation vary with y_0 on a linear scale. As can be seen from Figure S8, the peak-intensity only attenuates by 52%, even when the separation increases to 5 μm. Furthermore, such strong robustness ensures that even a micrometer-scale separation distance will not degrade the performance of particle detection but will effectively minimize the interaction between the external electromagnetic field from electrodes and the moving particle.

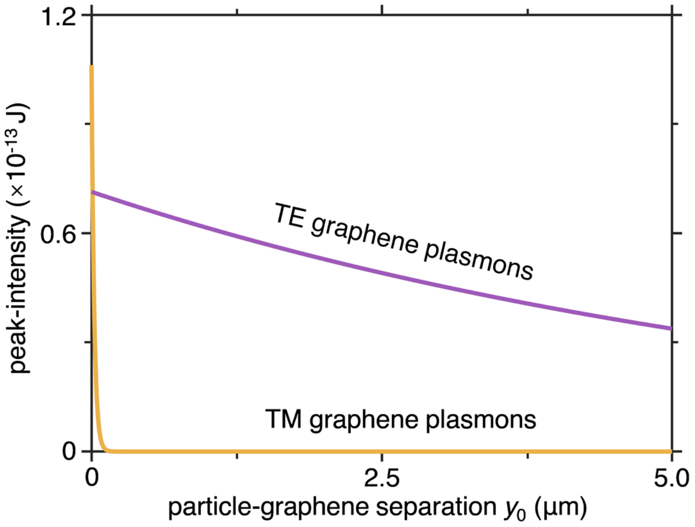

Figure S8 | The radiation peak-intensity as a function of particle-graphene separation y_0 on the order of micrometers. Other parameter setups are same as those in Figure 5.

Section S9. Detection scheme of TE graphene plasmon Cherenkov radiation.

To facilitate future experiments, we schematically show the possible experimental realization for detecting graphene plasmon Cherenkov radiation with the position sensitive photodetector array and grating technology in Figure S9. To be specific, the momentum compensation $\Delta k_z = 2\pi g/G$ from the grating (with grating period G) enables the radiation propagating along the y-direction to be extracted into free space, where g denotes the diffraction order. In this case the measured angle θ would be such that $\theta = \arctan \frac{\tan\theta'\left(k_z - \frac{2\pi g}{G}\right)}{k_z}$, where θ' is the free-space emission angle to be determined.

Remarkably, our work has the potential for on-chip integration. This is attributed to the following two key factors. First, the core structure of the device is compatible with micro-nano processing technologies. Second, the detectable range of the device could be flexibly adjusted by on-chip electrical control.

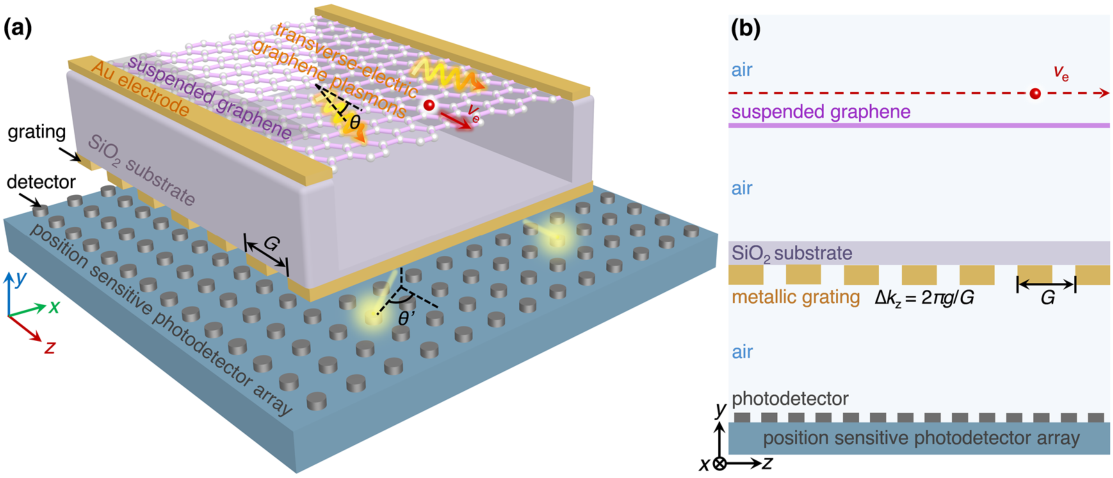

Figure S9 | Scheme for the detection of TE graphene plasmon Cherenkov radiation. (a) 3D view. The grating coupler is set at the bottom of the SiO_2 substrate. The TE graphene plasmons

generated from the charged particle are coupled to the free-space photons through the sub-wavelength grating coupler. Position sensitive photodetector array is applied to detect free-space photons in midinfrared to terahertz frequencies. (b) Side view. The momentum compensation from the grating is given by $\Delta k_z = 2\pi g/G$, where g is the diffraction order and G is the grating period.

<u>Section S10. More discussions on the low-index phonon polaritons operating in additional frequency band.</u>

Here, we reveal a type of TM surface phonon polariton with low mode refractive index in polar dielectric nanofilms, which can also be applied to particle detection in the ultra-high energy range. Taking silicon carbide (SiC) as an example (i.e., an air-SiC-air structure), we describe its main phonon modes via the single-oscillator Lorentz model, as follows:

$$\varepsilon_b(\omega) = \varepsilon_\infty + \frac{G\omega_0^2}{\omega_0^2 - \omega^2 - \mathrm{i}\gamma\omega} \tag{S31}$$

where $\varepsilon_b(\omega)$ is the dielectric function of SiC, ε_∞ is the high-frequency permittivity, G is the oscillator strength, ω_0 is the oscillator resonance frequency (i.e., transverse optical phonon frequency), and γ is the damping coefficient. By matching the boundary conditions for TM waves via Eq. (S3), we get the dispersion relation of TM surface phonon polaritons in an air-SiC-air structure as

$$\frac{\left(\frac{k_{y_b}}{\varepsilon_b} - \frac{k_{y_a}}{\varepsilon_a}\right)}{\left(\frac{k_{y_b}}{\varepsilon_b} + \frac{k_{y_a}}{\varepsilon_a}\right)} = e^{-\mathrm{i}k_{y_b}d_\mathrm{f}} \tag{S32}$$

where $k_{y_j} = \sqrt{\varepsilon_j k_0^2 - q^2}$, ε_a is the permittivity of air, and d_f is the thickness of the SiC film.

To demonstrate that the TM surface phonon polaritons supported by SiC nanofilms possess a

near-unity mode refractive index, we plot the dispersion relations of the coupled odd mode (solid line) and even mode (dashed line) for an air-SiC-air structure with different film thicknesses in Figure S10.

Notably, odd modes reflect the interesting property that upon decreasing SiC film thickness, the mode refractive indices n_{mode} of the coupled TM surface phonon polaritons gradually close to unity. For instance, at the operating frequency of f' = 27.8 THz, as the film thickness d_{f} varies from 150 nm, 50 nm, to 10 nm, the mode refractive index n_{mode} changes from 1.008, 1.0008, to 1.00004.

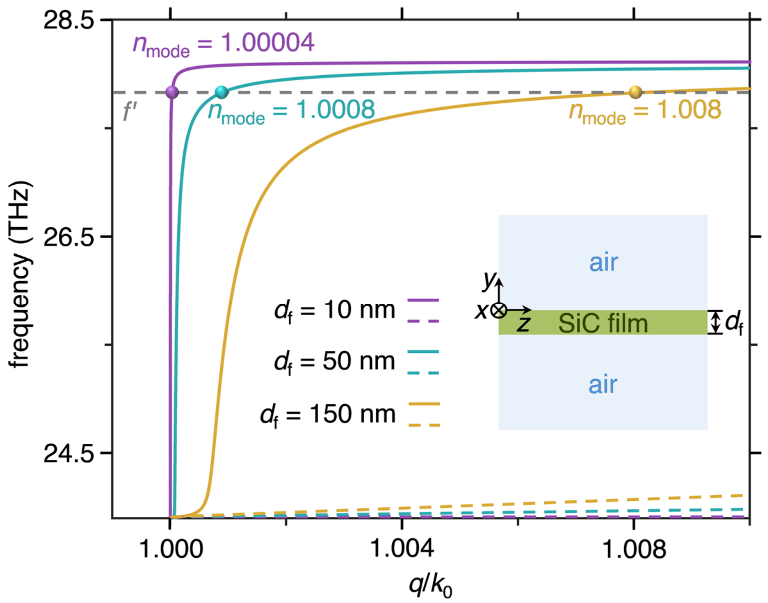

Figure S10 | Dispersion relation of TM surface phonon polaritons supported by an air-SiC-air structure under different thicknesses of the SiC film. The studied thickness d_{f} is 10 nm, 50 nm and 150 nm, respectively. The adopted parameters are as followings: $\varepsilon_{\infty} = 6.5$, G =2.5, ω_0 = 23.9 THz, γ = 0.2 THz, and f' = 27.8 THz.

Supplementary References

[1] F. H. L. Koppens, D. E. Chang, F. J. García de Abajo, *Nano Lett.* **2011**, 11, 3370.

[2] V. L. Ginzburg, V. N. Tsytovich, *Phys. Rep.* **1979**, 49, 1.
[3] H. Hu, X. Lin, J. Zhang, D. Liu, P. Genevet, B. Zhang, Y. Luo, *Laser Photonics Rev.* **2020**, 14, 2000149.
[4] S. Scheel, S. Buhmann, *Acta Phys. Slovaca* **2008**, 58, 675.
[5] F. J. García de Abajo, *Rev. Mod. Phys.* **2010**, 82, 209.
[6] N. Rivera, I. Kaminer, Nat. Phys. **2020**, 2, 538.
[7] Adam, I. et al., *Nucl. Instrum. Meth. A* **2005**, 538, 281.
[8] Kalicy, G. et al. (EIC PID Collaboration), *JINST* **2020**, 15, C11006.
